# Effect of outdoor games among school children in Northern Gujarat, India

**DOI:** 10.6026/97320630018791

**Published:** 2022-09-30

**Authors:** N Sivasubramanian, B Mahalakshmi, Sandeep Garg, Patel Shahin Aiyubdaud, Bepari Soma, KJ Shaijo, Robin Abraham, Bhasara Kalpana Ramji

**Affiliations:** 1 Department of Psychiatric Nursing, Nootan College of Nursing, Sankalchand Patel University, Visnagar, Gujarat - 384315, India; 2 Department Paediatric Nursing, Nootan College of Nursing, Sankalchand Patel University, Visnagar, Gujarat - 384315, India; 3 Tantia University, Sri Ganganagar, Rajasthan, India; 4 Sankalchand Patel University, Visnagar, Gujarat - 384315, India

**Keywords:** Act out method, school age children, outdoor games

## Abstract

Outdoor play works as an important tool for the children education. Providing a natural learning environment for children helps them to have an active and fulfilling life. Also, higher levels of attention and well-being are promoted when children play in green outdoor spaces. The importance of play for children's healthy development is grounded in a strong body of research. The study used an experimental research methodology, and data was obtained from 60 school-aged children using a purposive sample strategy and a checklist. The mean, standard deviation, and chi square test were used to analyze the data. After giving act out method, majority (85%) of the school age children had adequate knowledge, 15% of the school children had moderate knowledge regarding outdoor games and its importance. In data analysis the mean of pretest score was 6.43 and mean of post test score was 15.88. The mean difference was 9.45. Post test mean was more than pretest mean, which indicate the effectiveness of actout method on deficiency of outdoor game among school children. The standard deviation of pretest knowledge score was 3.9; the post test knowledge score was 2.47. The computed 't' value was 16.1, the DF was 59, and the P value was 1.67, all of which are significant. Religion, monthly income, and the age of the children all had an impact on the calculated chi square value. According to the findings of this study, the act out method was successful in boosting understanding about the lack of outdoor games among school-aged children.

## Background:

The unique characteristics and stimuli of the outdoor world provide a variety of play opportunities that are difficult to duplicate indoors [1]. Children benefit from being exposed to sunlight, natural elements, and open air while playing outside, which helps with bone development, immune system strength, and physical activity [2]. Also, higher levels of attention and well-being are promoted when children play in green outdoor spaces [3]. Outdoor time is linked to increased levels of physical activity. Physical activity delivers a myriad of health and wellbeing benefits for youngsters who engage in it on a regular basis [4]. Spending time outdoors and engaging in activities such as play has been shown to promote emotional and social resilience in children, in addition to the relevance of Physical Activity for children's health and wellness [5]. A growing body of research has looked into the health effects of children who engage in outdoor play. For example, several studies have discovered that children's mental health, physical activity, academic achievement, social development, and cognitive development all improve when they participate in sports [6]. Physical activity, according to some studies, has a positive impact on cognition, as well as brain structure and function. Physical activity, fitness, cognition, and academic achievement all had positive correlations [7]. Physical activity may have beneficial benefits on cognitive development during early childhood, according to a comprehensive analysis of natural play activities [8]. Due to the restriction of outdoor activities during Covid lockdown, children of all ages have become overly reliant on digital gadgets, putting them at risk for ocular disease and future myopia. Furthermore, constraints on outdoor movement have increased the amount of time spent on these gadgets for recreational purposes, such as playing video games and using social media. Although there is enough evidence to think that prolonged use of digital devices has an influence on ocular health in people of all ages, school-aged children in their formative years are the hardest harmed. This trend could lead to long-term behavioral changes with psychological and physiological consequences [9]. Competitive video gaming has been linked to lower levels of prosocial conduct in youngsters who play video games frequently. Competitive gaming on a regular basis may be linked to a decline in social behavior [10]. According to experts, a lack of outdoor play can cause health problems, emotional consequences, and even social skills impairment in children. According to Dr. Vipul Singh, a psychiatrist at GSVM Medical College, the main disadvantage of children's lack of outdoor play is behavior problems. Children have a lot of stamina, which they must channel into play. Children under the age of 16 must participate in an outdoor sport for at least 45 minutes each day, whether they are boys or girls [11]. There is a need to enhance public knowledge about children's right to play outside, as well as its benefits to their health, learning, and development. Recognizing the amount of time children spend in educational settings, concerns about having enough time and space to play outside should be factored into school planning and intervention [3]. Acting approaches, according to research, are effective in raising children's awareness. Because they are compatible with children's specific age characteristics, act out methods like drama or role play treatments are regarded effective ways for educating children. Acting out is a low-cost strategy that can have a significant impact on children's education by removing boredom from the learning process and assisting youngsters in preparing for adulthood [12]. Therefore, it is of interest to report the effect of act out method in improving school children’s awareness regarding deficiency of outdoor games.

## Methodology:

The study used an experimental research methodology, and data was obtained from 60 school-aged children from selected villages of Mehsana District, North Gujarat. Purposive sampling was used to choose 60 school-aged children based on the inclusion criteria. A knowledge questionnaire was given to each participant to assess their level of awareness about the lack of outdoor games prior to the test. After the pre test, act out method on Outdoor games was administered to samples through socio drama. The selected members from samples were trained to perform a situational act which describes the benefits of outdoor games and deficiency of outdoor activities. The concept, importance and deficiency of outdoor games were communicated to children through act out method. After 15 days of act out method, the samples were again assessed for the level of awareness on deficiency of outdoor games. After that, descriptive and inferential statistics such as mean, standard deviation, and chi square test were used to assess the pretest and post test data.

## Results:

Data given in Table 1 shows that majority (41.66%) of samples were belongs to age group 8-9 years. Majority (60%) of them were girls. Most of the children's parents had higher secondary and above level of education. Most of the children belong to nuclear family and residing in rural areas. After giving act out method as an intervention, majority (85%) of the school age children had gained adequate knowledge, 15% of the school children had moderate knowledge. Figure 1 show that prior to the application of the Act out approach, 75% of the participants in the pre-test lacked sufficient knowledge. There was a significant improvement in the sample's knowledge in the post-test, with 15% gaining moderate knowledge and 85% gaining adequate knowledge. In data analysis the mean of pretest score was 6.43 and mean of post test score was 15.88. The mean difference was 9.45; post test mean was more than pretest mean, which indicate the effectiveness of act out method on deficiency of outdoor game among school age children. The standard deviation of pretest knowledge score was 3.9; the post test knowledge score was 2.47. The calculated 't' value is 16.1 the DF value was 59 and P value was 1.67 that is significant. The data presented in Table 2 shows that the mean post-test level of school children (15.88±2.47) was lower than the mean pre-test level of school children (6.431 ± 3.9). The calculated 't' value (16.1) was greater than the table value (1.67) at 0.05 level of significance that shows the Act out method was effective in increasing the knowledge of outdoor game regarding school children. Demographic variables of sample such as age, gender, parent's education, occupation, type of family, number of children in family, area of residence etc. were assessed for each sample. Relationship of these variables with their level of awareness regarding outdoor games was analyzed using Chi square test. Chi-square analysis revealed that there was association of the level of knowledge with parent's education and area of residence. All other variables were not significant with level of knowledge.

## Discussion:

As per the current trends in health hazards of school age children, it is essential for them to understand the adverse effects of deficiency of outdoor games. The present study revealed that 75 % of children had inadequate knowledge regarding outdoor games. This was an indicator for the requirement of health education to school age children regarding the deficiency of outdoor games. The present study has determined that act out method is an effective teaching method which can be used to boost the knowledge of school children. A study conducted by Singh also supports the present finding. Singh's study conducted in 2019 at Odisha concluded that role playing method was the best method to teach children [13]. A study report by Shilpa & Swamy also revealed the effectiveness of acting method in improving the knowledge of children. Their study aimed to evaluate the effectiveness of role play in improving knowledge of school children regarding oral hygiene. The t test analysis revealed that there was a significant difference in pre-test and post-test which assured the effectiveness of role play as a teaching method [15]. Act out method is a way of interaction with children using social drama or role plays in-order to convey the important messages to their brain. As this method can greatly achieve the attention and concentration of children, it eliminates the possibility of boredom like in lecture method. In the association of knowledge with selected demographic variables, the present study found out that children’s awareness on outdoor games is closely associated with their parent's education status. This finding is supported by the report of Sabhya Samiali [14]. Their study findings revealed that education status of parents was associated with level of knowledge of school going children regarding benefits of outdoor games. Another study conducted by Shilpa also supports this finding by concluding that parents educational status influences children's awareness significantly [15]. The value of outdoor play activities in children's health is clearly understood by knowledgeable parents. They will provide the facilities and motivate the children for indulging in outdoor games. The children of such parents will have moderate knowledge and awareness regarding importance of outdoor games.

## Conclusion:

Data shows that the act out method was successful in boosting understanding about the lack of outdoor games among school-aged children. As a result of COVID-19 lockdown, the outdoor activities by the children decreased significantly by the last 2 years. This has raised the addiction to indoor games using smart phones and video games. Many of children and parents were not aware about the dangerous effects of avoiding outdoor games by children. Children need much attention and motivation in this aspect. Thus, effective methods to educate the children to improve their awareness on benefits and deficiency of outdoor games are needed. Hence, data strongly recommends the use of act out method as an effective teaching strategy for the school age children.

## References

[1] Stephenson A (2002). Eur Early Child Educ Res J.

[2] Dyment JE, Bell AC (2008). HealthEduc Res.

[3] Bento G, Dias G (2017). Porto Biomed J..

[4] Aggio D (2017). Prev Med Rep..

[5] Herrington S, Brussoni M (2015). CurrObes Rep..

[6] Dankiw KA (2020). PLoS One..

[7] Donnelly JE (2016). Med Sci Sports Exerc..

[8] Carson V (2016). J Sci Med Sport..

[9] Saxena R (2021). Indian J Ophthalmol..

[10] Lobel A (2017). J Youth Adolesc..

[12] Soleymani MZ (2017). J Educ Health Promot..

[13] Singh S (2019). Adv Practice Nurs.

[14] Samiali S, Thakur S (2021). Int J Sci and Res (IJSR).

[15] Shilpa PM, Swamy PGN (2015). IOSR Journal of Nursing and Health Science.

